# Mixed features and suicide attempts in youth depression: a six-month follow-up study

**DOI:** 10.1186/s12888-026-07783-x

**Published:** 2026-01-14

**Authors:** Kunrong Lin, Yuhang He, Jie Zhang, Yufen Ou, Hongbo He

**Affiliations:** 1 The Affiliated Brain Hospital, Guangzhou Medical University, Guangzhou, China; 2 Guangdong Engineering Technology Research Center for Translational Medicine of Mental Disorders, Guangzhou, China; 3 Guangdong Mental Health Center, Guangdong Provincial People’s Hospital (Guangdong Academy of Medical Sciences), Southern Medical University, Guangzhou, China

**Keywords:** Major depressive episode, Mixed features, Suicide attempt, Adolescents, Prospective cohort

## Abstract

**Background:**

Mixed features during major depressive episodes (MDE) may confer elevated suicide risk, yet evidence in adolescents and young adults remains limited. This study aimed to investigate the association between mixed features and suicide attempts in this population.

**Methods:**

We conducted a prospective cohort study including 951 adolescents and young adults (aged 13–25 years) with MDE, recruited from multiple hospitals in South China (2022–2023). Mixed features were defined as ≥ 3 (hypo)manic symptoms during depression. Baseline suicidal risk was assessed using item 3 of the Hamilton Depression Rating Scale (HAMD-17). Follow-up suicide attempts were assessed at 1, 3, and 6 months via structured interviews. Cox proportional hazards models evaluated time to first suicide attempt, with multiple imputation applied for missing data. Subgroup analyses examined effect modification by sex, episode status, age, and illness duration.

**Results:**

Of 951 participants, 316 (33.2%) met criteria for mixed features. Participants with mixed features had a higher incidence of suicide attempts during follow-up, reaching significance at month 6 (*p* = 0.032). Cox regression showed that mixed features were associated with an increased hazard of suicide attempts (HR = 1.32, *p* = 0.045; complete-case analysis HR = 1.89, *p* = 0.039). Baseline depressive severity and suicidal ideation independently predicted risk. Subgroup analyses showed a significantly stronger effect among participants in their first depressive episode. Participants with shorter illness duration also exhibited the highest hazard ratio, although the interaction did not reach conventional significance.

**Conclusions:**

Mixed features were associated with an increased risk of suicide attempts in adolescents and young adults with depression, with the effect being most evident in first-episode patients. Systematic assessment of mixed features may help identify individuals who would benefit from early and tailored preventive strategies.

**Clinical trial number:**

Not applicable.

**Supplementary Information:**

The online version contains supplementary material available at 10.1186/s12888-026-07783-x.

## Introduction

Major depressive disorder (MDD) ranks among the leading causes of global disability, owing to its high prevalence and profound impact on psychosocial functioning, and it disproportionately affects adolescents and young adults [1–3]. Among young individuals with depression, suicidal behavior has become a major public health concern. Recent global evidence indicates that suicide is currently the second leading cause of death among those aged 15–29 years [4]. Suicide attempts, as a more frequent manifestation of suicidal behavior compared with suicide deaths, also serve as a strong predictor of subsequent suicide. In a recent study involving psychiatric inpatients, approximately 22% of adolescents aged 13–17 years and 17.9% of young adults aged 18–25 years reported suicide attempts within six months prior to hospitalization, with depressive disorders emerging as one of the strongest predictors of such behavior [5]. These findings underscore the critical importance of identifying clinical features that confer elevated risk for suicidal behavior among young patients with depression, in order to facilitate early intervention and reduce suicide attempts.

Depression with mixed features refers to the presence of three or more (hypo)manic symptoms during a major depressive episode (MDE), in the absence of a full manic or hypomanic episode. According to the Diagnostic and Statistical Manual of Mental Disorders, Fifth Edition (DSM-5) [6], nonspecific symptoms such as irritability, which may occur in both manic and depressive states, should be excluded from this specification [7, 8]. Depressive episodes with mixed features can occur across various psychiatric disorders, including MDD, bipolar disorder, and schizoaffective disorder. Meta-analytic data indicate that approximately 11.6% of all major depressive episodes meet the DSM-5 criteria for mixed features [9]. Among adults aged 18 years and older with MDD, epidemiological evidence from the United States further shows that about 15.5% experience mixed features during depressive episodes [10]. Although epidemiological data on mixed features among patients with MDD under the age of 18 remain limited, previous studies have suggested that mixed features tend to occur more frequently in those with an earlier age of onset [11]. Given that this clinical presentation may be particularly common among younger individuals with MDD, investigating mixed features in this population is of considerable importance.

One major reason is that mixed features have often been linked to an increased risk of suicidal behavior. For example, a community-based study among young adults aged 18–24 years found that individuals experiencing concurrent (hypo)manic and depressive symptoms were more than twice as likely to have a history of suicidal behavior compared with those with depressive episodes alone [12]. Further research has shown that the presence of manic symptoms during follow-up visits among patients with MDD significantly increased the risk of suicide attempts; however, this association became nonsignificant when both suicidal ideation and attempts were analyzed as joint outcomes [13]. Recent cross-sectional studies in adult populations have further supported these findings, indicating that mixed features were not significantly associated with suicidal ideation but were closely linked to a markedly increased risk of suicide attempts [14]. Taken together, these findings may help to explain the higher prevalence of suicide attempts observed among individuals aged 13–25 years, suggesting that the presence of mixed features may substantially elevate the risk of suicide attempts during major depressive episodes in patients with MDD.

However, although mixed features may be more prevalent among adolescents and young adults, longitudinal studies focusing on these age groups remain scarce [9]. Reliance on cross-sectional designs or studies conducted in adult populations makes it difficult to clarify the suicide attempt risk associated with mixed features. Furthermore, most existing studies on depression with mixed features have focused primarily on current depressive episodes, without adequately differentiating diagnostic categories. Given that diagnostic heterogeneity may influence the manifestation of MDE, it is necessary to investigate the association between mixed features and suicide attempts specifically among patients with MDD. Such efforts may help to clarify the predictive value of mixed features for subsequent suicide risk and inform the development of targeted prevention strategies. Finally, mixed features may interact with various clinical and demographic factors (e.g., sex and illness duration), and their underlying mechanisms warrant further investigation [15–17].

To address these research gaps, we conducted a 6-month prospective cohort study involving adolescents and young adults diagnosed with MDD who were currently experiencing a MDE. The primary aims were to examine whether mixed features could predict [1] the timing of the first suicide attempt during follow-up, and [2] differences in suicide risk across subgroups defined by factors such as sex and illness duration.

## Methods

### Participants

Between January 2022 and December 2023, a total of 1,631 individuals were screened for eligibility across multiple tertiary hospitals, including two specialized psychiatric hospitals (the Affiliated Brain Hospital of Guangzhou Medical University and Shenzhen Kangning Hospital) and three general tertiary hospitals (Guangdong Mental Health Center, the Fifth Affiliated Hospital of Sun Yat-sen University, and Guangdong Provincial Hospital of Traditional Chinese Medicine). Both inpatients and outpatients were consecutively recruited from the departments of psychiatry, psychology, or sleep medicine. Of these, 680 individuals were excluded for not meeting inclusion criteria, including ineligible age, absence of a current MDE, diagnosis of bipolar disorder or primary psychotic disorder, or incomplete baseline demographic or clinical assessments. The final analytic sample included 951 patients with depressive disorders, of whom 316 met DSM-5 criteria for mixed features.

Recruitment and clinical assessments were conducted by trained research staff, including psychiatrists and research assistants with professional backgrounds in psychiatry. The research team also included experienced child and adolescent psychiatrists, and most investigators had completed at least a three-month clinical rotation in child and adolescent psychiatry. For participants under 18 years of age, recruitment, diagnostic interviews, and assessments were conducted or supervised by these child and adolescent psychiatrists to ensure diagnostic accuracy, age-appropriate communication, and adherence to ethical standards for minors.

**Fig. 1:**
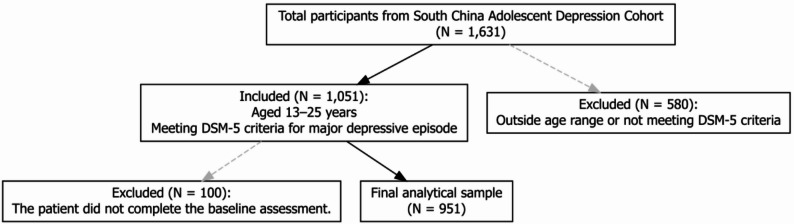
Participant inclusion and exclusion flow diagram

Inclusion criteria comprised the following: (1) age between 13 and 25 years; (2) a diagnosis of MDD according to DSM-5 criteria, with participants currently experiencing a first-episode or recurrent MDE; and (3) availability of complete baseline data. Exclusion criteria were as follows: (1) failure to meet DSM-5 criteria for MDE or age outside the eligible range; (2) Participants with any current diagnosis of bipolar disorder, schizophrenia, or other primary psychotic disorders according to DSM-5 criteria were excluded; (3) cognitive impairment or clinically diagnosed organic brain disease; and (4) missing baseline demographic or Clinical assessments (Fig. 1).

The study protocol was approved by the Ethics Committee of the Affiliated Brain Hospital of Guangzhou Medical University (Approval No. 2022-031). Written informed consent was obtained from all participants; for minors, additional consent was obtained from a parent or legal guardian.

### Measures

#### Demographic and clinical characteristics

Baseline demographic and clinical characteristics included age, gender, student status, romantic relationship status, illness duration, episode status (first-episode or recurrent), family psychiatric history, and lifestyle factors, including smoking and alcohol use. Categorical variables were summarized as counts and percentages.

#### Mixed features

Diagnostic interviews were conducted in a semi-structured format based on DSM-5 criteria. All interviewers were psychiatrists with experience in child and adolescent psychiatry or trained research staff. They received standardized training and supervision prior to data collection to ensure diagnostic consistency. Although no formal inter-rater reliability assessment was performed, standardized training and regular supervision were implemented to maintain procedural uniformity. Each interview followed a standardized sequence: (1) Confirm the presence of a current MDE; (2) Exclude affective disorders with psychotic features, schizophrenia spectrum and other psychotic disorders, and any history of manic or hypomanic episodes; (3) Assess the DSM-5 “with mixed features” specifier, defined as the presence of three or more manic/hypomanic symptoms (elevated or expansive mood, inflated self-esteem, pressured speech, flight of ideas, increased energy or goal-directed activity, engagement in risky behaviors, or decreased need for sleep) on most days of the depressive episode. Individuals meeting these criteria were classified as having an MDE with mixed features, whereas those meeting criteria for MDD but not the mixed features specifier were categorized as having a non-mixed MDE.

#### Suicide attempts

Baseline suicide risk was assessed using item 3 of the 17-item Hamilton Depression Rating Scale (HAMD-17) [18], which ranges from 0 to 4: 0 = no suicidal thoughts; 1 = feels life is meaningless; 2 = wishes to be dead or frequently thinks about death; 3 = passive suicidal thoughts; 4 = severe suicidal behavior. Participants were categorized into baseline suicide risk groups according to this item, whereas the total score of the remaining items reflected baseline depressive severity independent of suicidal risk [19, 20].

Follow-up suicide attempts were assessed at 1, 3, and 6 months through scheduled telephone interviews, with information obtained from both participants and their family members. The interview included three questions: (1) whether a suicide attempt had occurred between the follow-up intervals; (2) if yes, the specific behaviors involved; and (3) the immediate clinical consequence of the attempt (e.g., injury severity or need for medical treatment).

### Procedure

Baseline assessments were performed through structured interviews conducted by trained psychiatrists. Follow-up assessments of suicide attempts were conducted at 1, 3, and 6 months as outlined above.

### Statistical analysis

#### Missing data

During the 6-month follow-up, 718 participants completed the 1-month assessment, 628 completed the 3-month assessment, and 502 completed the 6-month assessment (Supplementary Figure). Participants who attempted suicide during follow-up were not assessed further, as the suicide attempt constituted the primary outcome. Although Little’s MCAR test indicated that data were not missing completely at random (*p* < 0.05), logistic regression analyses showed that missingness was primarily associated with observed baseline characteristics such as younger age, higher baseline depressive severity, and shorter illness duration (Supplementary Table 1). These findings supported the Missing At Random (MAR) assumption. Multiple imputation (MI) was performed in the full study sample, with follow-up suicide attempt data imputed for participants with incomplete follow-up. Under this assumption, missing values for suicide attempts at the 1-, 3-, and 6-month follow-ups were handled using multiple imputation by chained equations (MICE, version 3.17.0) [21], following the procedures described by Austin et al. [22]. The three follow-up suicide attempt variables were imputed using the logistic regression method (“logreg”), which is appropriate for binary outcomes. The imputation model included baseline predictors that were theoretically and empirically associated with missingness or suicide risk, including age, gender, illness duration, smoking history, family history, baseline depression severity, and lifetime suicide history. Five imputed datasets were generated, each with 50 iterations and a random seed of 500. Covariates without missing data were not imputed. All statistical analyses were conducted separately within each imputed dataset, and estimates were pooled using Rubin’s rules [23]. Complete-case analyses (CCA; *n* = 459), restricted to participants with complete follow-up data, were also conducted to confirm the robustness of the results.

#### Kaplan–Meier survival analysis

Time to first suicide attempt during the 6-month follow-up was initially analyzed using Kaplan–Meier curves. Survival curves were generated separately for the complete-case dataset and for the multiple imputation (MI) datasets to account for missing data. Group differences were evaluated using the Log-rank test, and corresponding P values were reported.

#### Cox proportional hazards regression

Time to the first suicide attempt during the 6-month follow-up was analyzed using Cox proportional hazards regression. The primary exposure variable was the presence of mixed features. Covariates included gender, age, episode status, illness duration, baseline depressive severity excluding the suicide item (HAMD-17 without item 3), baseline suicidal ideation (HAMD-17 item 3), and history of suicide attempt. Covariates were selected based on two considerations. First, variables theoretically or empirically associated with suicidal behavior (i.e., depressive severity, baseline suicidal ideation, prior suicide attempts, gender, and age) were included to reduce potential confounding. Second, variables showing significant baseline group differences (i.e., episode status and illness duration) were additionally controlled for, as they might confound the association between mixed features and suicide attempts. This approach aimed to balance the control of potential confounders with model parsimony and stability. Hazard ratios (HRs) and 95% confidence intervals (CIs) were estimated. Proportional hazards assumptions were assessed using Schoenfeld residuals, and stratified models were applied when necessary [24]. Separate Cox models were fitted to each imputed dataset, and estimates were pooled according to Rubin’s rules. Sensitivity analyses were conducted using complete-case data. Although latent growth curve models (LGCM) [25] and recurrent event survival models [26] could theoretically be applied, they were not suitable for the present study due to the data structure. Specifically, only the occurrence of suicide attempts was longitudinally assessed at 1, 3, and 6 months, while other key variables (e.g., mixed features and depressive severity) were measured only at baseline. Moreover, suicide attempts were relatively infrequent in this cohort, and modeling recurrent events could result in unstable estimates. Therefore, the Cox proportional hazards model focusing on time to first suicide attempt was deemed the most appropriate analytic approach.

#### Subgroup analyses

Stratified Cox regression analyses were conducted within each imputed dataset to examine the consistency of effects across key clinical subgroups, including gender, episode status, illness duration, age group (adolescents vs. adults). Interaction terms between mixed features and subgroup variables were tested. Pooled hazard ratios, 95% confidence intervals, and interaction P values were reported. Forest plots were used to visualize subgroup-specific effects.

All analyses were performed using R version 4.4.2. Statistical significance was set at two-tailed *p* < 0.05. The study adhered to the guidelines of the International Committee of Medical Journal Editors (ICMJE) and the Strengthening the Reporting of Observational Studies in Epidemiology (STROBE).

## Results

### Baseline characteristics

Baseline sociodemographic and clinical characteristics stratified by mixed-feature status are shown in Table 1. Participants with mixed features were slightly younger than those without mixed features (t = 2.22; *p* = 0.026), and a higher proportion were aged < 18 years (χ² = 6.17; *P* = 0.013). The mixed-features group had a greater proportion of females (χ² = 12.1; *p* = 0.001). No significant differences were observed in student status or romantic relationship status.

**Table 1:** Baseline sociodemographic and clinical profile by mixed features status

Variable	MDE With Mixed Features(*n* = 316)	MDE Without Mixed Features(*n* = 635)	Stat	*P* value
Age	18.01 ± 3.06	18.49 ± 3.27	t = 2.22	0.026
Age, n (%)			χ² = 6.17	0.013
< 18y	161 (50.9%)	268 (42.2%)		
≥ 18y	155 (49.1%)	367 (57.8%)		
Gender, n (%)			χ² = 12.1	0.001
Male	51 (16.1%)	168 (26.5%)		
Female	265 (83.9%)	467 (73.5%)		
Student status, n (%)			χ² <0.001	0.997
Non-Students	59 (18.7%)	117 (18.4%)		
Student	257 (81.3%)	518 (81.6%)		
Romantic relationship, n (%)			χ² = 0.01	0.911
Yes	60 (19.0%)	124 (19.5%)		
No	256 (81.0%)	511 (80.5%)		
Illness duration, n (%)			χ² = 20.62	< 0.001
< 6 months	38 (12.0%)	128 (20.2%)		
6–24 months	111 (35.1%)	264 (41.6%)		
> 24 months	167 (52.8%)	243 (38.3%)		
Episode status, n (%)			χ² = 24.13	< 0.001
First episode	129 (40.8%)	368 (58.0%)		
Recurrent episode	187 (59.2%)	267 (42.0%)		
Family history, n (%)			χ² = 2.23	0.136
Yes	73 (23.1%)	119 (18.7%)		
No	243 (76.9%)	516 (81.3%)		
Smoke history, n (%)			χ² = 2.45	0.118
Yes	49 (15.5%)	74 (11.7%)		
No	267 (84.5%)	561 (88.3%)		
Alcohol history, n (%)			χ² = 1.72	0.19
Yes	28 (8.9%)	40 (6.3%)		
No	288 (91.1%)	595 (93.7%)		
Suicide history, n			χ² = 22.65	< 0.001
Yes	157 (49.7%)	214 (33.7%)		
No	159 (50.3%)	421 (66.3%)		
HAMD-17 total	19.55 ± 7.63	19.63 ± 7.39	t = 0.146	0.884
Severity of depression			χ² = 0.380	0.538
Mild to moderate	230 (72.8%)	474 (74.6%)		
Severe	86 (27.2%)	161 (25.4%)		
HAMD-17 no suicide	17.2785 ± 6.83	17.4992 ± 6.99	t = 0.477	0.634
HAMD-17 suicide	2.27 ± 1.36	2.13 ± 1.34	t = -1.553	0.121

Clinically, participants with mixed features were more likely to have an illness duration > 24 months (χ² = 20.62; *p* < 0.001), recurrent episodes (χ² = 24.13; *p* < 0.001), and a history of suicide attempts (χ² = 22.65; *p* < 0.001). There were no significant group differences in family psychiatric history, smoking history, or alcohol use. Baseline depressive severity, measured by HAMD-17 total score excluding item 3, did not differ between groups (t = 0.477; *p* = 0.634), and baseline suicidal risk, assessed by HAMD-17 item 3, was comparable (t = -1.553; *p* = 0.121).

### Follow-up suicide attempts

Follow-up suicide attempts during the 6-month observation period are summarized in Table 2. At month 1, incidence did not differ between groups (χ² = 0.01; *p* = 0.905). By month 3, the proportion of participants reporting a suicide attempt was greater in the mixed-features group (χ² = 3.35; *p* = 0.067), approaching statistical significance. At month 6, the mixed-features group had a significantly higher incidence of attempts (χ² = 4.62; *p* = 0.032). All counts and percentages are based on raw (non-imputed) data.

**Table 2:** Follow-up suicide attempts by mixed features status at 1, 3, and 6 months

Timepoint	No Mixed Features, *n* (%)	Mixed Features, *n* (%)	χ²	*p* value
Month 1	24 (5.3%)	13 (5.9%)	0.01	0.905
Month 3	16 (3.7%)	15 (7.5%)	3.35	0.067
Month 6	12 (3.6%)	14 (8.5%)	4.62	0.032

Note. Values are based on raw (non-imputed) data

**Fig. 2:**
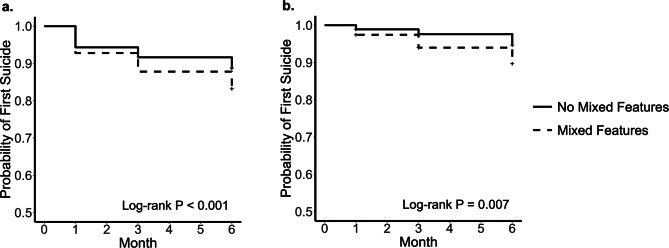
Kaplan–Meier survival curves for suicide attempt during the 6-month follow-up: complete-case analysis and multiple imputation. Note. (**a**) Kaplan–Meier survival curve based on multiple imputation, illustrating time to first suicide attempt during the 6-month follow-up, with missing data handled using multiple imputation. (**b**) Kaplan–Meier survival curve based on complete-case analysis, illustrating time to first suicide attempt during the same follow-up period

Consistent with these cross-sectional comparisons, Kaplan–Meier survival curves demonstrated a significantly faster decline in survival probability (i.e., time to first suicide attempt) among participants with mixed features. This difference was evident both in the multiply imputed analyses (log-rank test, *p* < 0.001) and in complete-case analyses (*p* = 0.007), underscoring the robustness of the findings (Fig. 2).

### Multivariable survival analyses

Results from multivariable Cox regression models are presented in Table 3. After adjustment for demographic and clinical covariates, mixed features remained significantly associated with an increased risk of suicide attempts during follow-up. Specifically, individuals with mixed features had a 32% higher hazard in the multiple imputation analysis (HR = 1.32, *p* = 0.045) and nearly doubled risk in the complete-case analysis (HR = 1.89, *p* = 0.039). A history of suicide attempts and greater baseline depressive symptom severity were also independently associated with elevated risk. Longer illness duration (> 24 months) was related to a lower hazard of suicide attempts. Other variables-including gender, age, and episode status-were not significantly associated with risk. Proportional hazards assumptions were satisfied. Although effect sizes differed slightly between the MI and CCA analyses, the direction and pattern of associations were consistent, supporting the robustness of the findings.

**Table 3:** Multivariable regression analyses for suicide attempts during follow-up

Variable	Multiple Imputation (MI)		Complete Case Analysis (CCA)	
	HR (95% CI)	*P* value	HR (95% CI)	*P* value
Mixed Features(Ref = No)				
Yes	1.32 (1.02–1.69)	0.045	1.89 (1.03–3.46)	0.039
Gender (Ref = Male)				
Female	1.28 (0.82–2.00)	0.314	0.86 (0.39–1.9)	0.708
Episode Status (Ref = First Episode)				
Recurrent episode	1.13 (0.80–1.59)	0.497	1.84 (0.9–3.75)	0.094
Illness duration(Ref = < 6 months)				
6–24 months	1.12 (0.76–1.66)	0.565	1.33 (0.49–3.58)	0.579
> 24 months	0.49 (0.29–0.82)	0.026	0.36 (0.11–1.13)	0.080
Suicide History (Ref = No)				
Yes	1.87 (1.39–2.52)	0.001	3.21 (1.58–6.52)	0.001
age	0.96 (0.92–1.00)	0.077	0.97 (0.88–1.08)	0.613
HAMD17 no suicide	1.04 (1.02–1.07)	0.003	1.06 (1.01–1.11)	0.017
HAMD17 suicide	1.27 (1.11–1.46)	0.004	1.21 (0.91–1.59)	0.188

Note. Hazard ratios (HRs) with 95% confidence intervals (CIs) are shown. Results are presented for both multiple imputation (MI) and complete-case analysis (CCA). Reference categories are indicated in the table

### Subgroup analyses

**Fig. 3:**
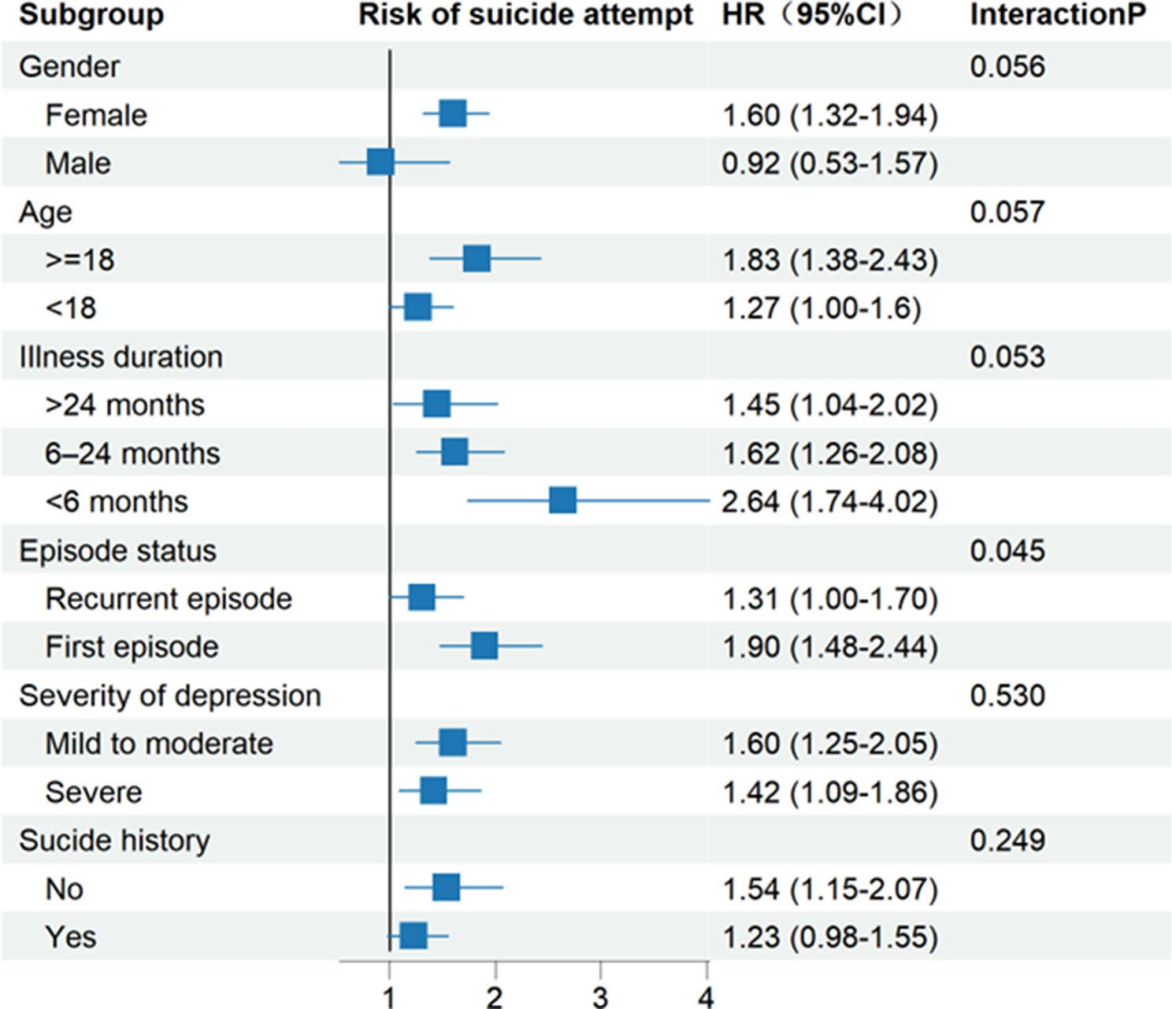
Forest plot of subgroup analyses for the association between mixed features and suicide attempt. Note. Forest plot showing the association between mixed features and the risk of first suicide attempt during the follow-up period across subgroups

Subgroup analyses revealed significant effect modification by episode status (interaction *p* = 0.045) and marginally significant interactions for illness duration (*P* = 0.053), gender (*p* = 0.056), and age (*p* = 0.057). The association between mixed features and risk of suicide attempt was strongest among participants with an illness duration of < 6 months (HR = 2.64) and those experiencing their first depressive episode (HR = 1.90). The effect was also more pronounced among female (HR = 1.60) and adult participants (HR = 1.83), although the corresponding interaction terms did not reach conventional statistical significance. No significant interaction effects were observed for depressive severity (*p* = 0.530) or suicide history (*p* = 0.249) (Fig. 3).

## Discussion

In this prospective cohort study involving Chinese adolescents and young adults with MDD, the presence of mixed features during a depressive episode was found to be significantly associated with an increased risk of suicide attempts over a six-month follow-up period. After controlling for demographic and clinical covariates, mixed features remained an independent predictor of suicide attempt risk. Further subgroup analyses indicated that patients experiencing their first depressive episode with mixed features were at a significantly higher risk of subsequent suicide attempts compared with those with recurrent episodes. Moreover, the influence of mixed features appeared to be most pronounced among participants with a shorter illness duration, whereas the associated risk was lowest among male patients. Taken together, these findings provide further evidence for the prognostic value of mixed features in adolescent and young adult depression. They also highlight the importance of early identification and timely intervention in high-risk subgroups, which may help inform clinical practice and suicide prevention strategies.

Consistent with previous research, our findings indicate that the presence of mixed features is significantly associated with an increased risk of suicidal behavior. As mentioned earlier, a large-scale study conducted in France among individuals experiencing depressive episodes found that those with mixed features exhibited a markedly higher risk of suicide compared with their non-mixed counterparts [14]. Similarly, a cross-sectional study from Brazil demonstrated that patients who experienced both depressive and mixed episodes were at increased risk of suicide [12]. Taken together, these converging findings suggest that the association between mixed features and suicide risk may represent a cross-cultural phenomenon. Nevertheless, given the substantial variations in personality traits, interpersonal contexts, and social support systems among adolescents and young adults across different cultural settings, further studies are warranted to confirm the generalizability of this relationship.

Subgroup analyses indicated that the association between mixed features and time to first suicide attempt during follow-up varied across clinical subgroups. The relationship was strongest among patients with a short illness duration (< 6 months) and those experiencing their first depressive episode, suggesting that mixed features in the early phase of depression may represent a particularly high-risk condition in young patients with MDD. Previous studies have also reported increased suicide risk among patients in their first episode or early course of illness [27], possibly reflecting limited illness awareness among patients and families or inadequate pharmacological treatment. Another possible explanation is that individuals exhibiting mixed symptoms early in the course of depression may have greater affective instability. However, because our study did not include repeated mood assessments during follow-up, we were unable to verify subsequent changes in clinical status. Future research should therefore focus on the timing and persistence of mixed features to clarify their dynamic influence on suicidal behavior. Although the interaction effects of sex and age only approached significance, the stronger association observed in female patients aligns with prior reports of higher suicide attempt rates among women. Moreover, the link between mixed features and suicide attempts appeared stronger in young adults (18–25 years) than in adolescents (13–17 years), possibly because adolescents already have a high baseline suicide risk [5], reducing the relative impact of mixed features. This interpretation is consistent with prior epidemiological findings and our Cox regression results showing a higher overall risk of suicide attempts among adolescents than young adults.

The mechanisms underlying the association between mixed features and suicide attempts remain incompletely understood. Prior research indicates that individuals with mixed features do not necessarily exhibit greater severity of suicidal ideation compared with those without such features [14]. This suggests that mixed features may facilitate the transition from suicidal thoughts to suicidal acts rather than intensifying ideation per se. Manic or hypomanic symptoms during depressive episodes are frequently characterized by diminished impulse control and affective instability, both of which may amplify emotional reactivity to transient stressors and lower the behavioral threshold for suicidal behavior [28, 29]. Consequently, depressive episodes accompanied by mixed features may confer heightened vulnerability to suicidal acts through these manic-like manifestations. From a neurobiological perspective, mixed states may arise from genetic susceptibility within the circadian rhythm and dopaminergic neurotransmission systems, as well as dysregulation of the catecholamine–acetylcholine balance, which collectively contribute to affective instability and behavioral disinhibition [30]. Taken together, these findings imply that mixed features may elevate suicide risk by accelerating the transition from suicidal ideation to action through neurobiologically driven emotional dysregulation and impaired impulse control.

Adolescents and young adults with major depressive disorder who exhibited mixed features showed an elevated risk of suicide attempts during the six-month follow-up period, underscoring the clinical importance of early recognition and intervention in this subgroup. Given their heightened vulnerability, developing preventive strategies tailored to individuals with mixed features represents a critical step toward reducing suicide risk among youth with depression. In this context, we conceptually propose that suicide prevention efforts may benefit from integrating the stepped-care model [31, 32] with risk stratification based on mixed features. Originally designed to guide intervention intensity according to illness severity, this framework could inform future research on tiered approaches, in which patients with mixed features might be prioritized for more intensive or specialized treatments. This proposal should be regarded as a conceptual implication rather than a data-driven recommendation, highlighting the need for subsequent interventional or implementation studies to test its practical applicability.

This study has several strengths. First, we included a relatively large and clinically well-characterized sample of adolescents and young adults with depression, integrating both baseline and longitudinal follow-up data. We also applied complete-case and multiple imputation analyses to ensure the robustness of our findings, while additionally conducting subgroup analyses to explore potential differences across populations. Second, the multicenter design, which covered several hospitals in South China, enhances the external generalizability of our results. However, several limitations should be acknowledged. First, the attrition rate at the 6-month follow-up was relatively high (47.2%), which may partly be attributable to school policies prohibiting smartphone use, parental work commitments, or voluntary withdrawal by participants who had recovered. Although both MI and CCA were applied as sensitivity analyses, some differences in the magnitude of effect sizes were observed. These discrepancies likely reflect differences in sample size and information retention rather than inconsistencies in the direction of effects. MI retains statistical efficiency by utilizing all available information under the MAR assumption, whereas CCA excludes participants with missing data, resulting in reduced sample size and wider confidence intervals. Overall, the consistency in the direction and pattern of associations across both approaches suggests that the influence of missing data on the study conclusions was minimal. Second, there is ongoing debate in clinical practice regarding the definition and diagnosis of mixed features, and our assessments were not administered strictly item by item in accordance with the Structured Clinical Interview for DSM-5 (SCID-5) [33]. This may limit comparability with other studies. Additionally, because this study was based on a hospital-recruited sample of adolescents and young adults rather than a population-based cohort, the relatively high proportion of mixed features (33.2%) observed may reflect selection bias inherent to clinical recruitment settings, which could limit the generalizability of our findings. Third, due to practical constraints inherent to large-scale data collection, several potentially important psychosocial and clinical variables were not systematically assessed or statistically controlled, including hopelessness [34]. Although hopelessness is commonly viewed as being more closely associated with suicidal ideation, longitudinal evidence suggests that it may also be relevant to suicide attempts. In addition, other factors such as current comorbidities [35], psychopharmacological treatment, illicit substance use [36], and adverse childhood experiences [37] may also influence subsequent suicide attempts and could have contributed to residual confounding. Future studies should aim to incorporate these important psychosocial and clinical variables to enable a more comprehensive understanding of suicide risk among youth with depression. Fourth, given the relatively small number of suicide attempt events in the sample and considering that a few participants died by suicide, we did not analyze repeated suicide behaviors during the follow-up period. In addition, although other major psychiatric disorders were excluded at baseline, some participants may have developed such conditions during follow-up, and therefore residual confounding cannot be entirely ruled out. Future research should replicate these findings in multi-center and cross-cultural settings and further explore neurobiological mechanisms using multimodal approaches, including neuroimaging, electrophysiology, and genetic profiling [38]. Such methods are well suited to distinguishing state- and trait-like neural processes and identifying convergent biomarkers related to suicidal vulnerability. Randomized controlled trials targeting affective instability and impulsivity are also warranted. Incorporating models such as the ideation-to-action framework [39, 40] may further clarify the transition from suicidal ideation to attempts and inform more timely, individualized prevention strategies.

In conclusion, our findings indicate that mixed features in adolescents and young adults with depression are significantly associated with an increased risk of suicide attempts over time. Systematic screening for mixed features at the initial clinical assessment—particularly among patients experiencing their first depressive episode with mixed symptoms—may be important for implementing early and targeted suicide prevention strategies. Early identification of this high-risk subgroup could facilitate timely treatment planning and potentially improve long-term outcomes among youth with major depressive disorder.

## Supplementary Information

Below is the link to the electronic supplementary material.

Supplementary Material 1

## Data Availability

Due to privacy restrictions and the ongoing nature of the study, the datasets generated and/or analyzed during the current study are not publicly available. However, they can be obtained from the corresponding author upon reasonable request.

## References

[1] Luo J, Tang L, Kong X, Li Y. Global, regional, and National burdens of depressive disorders in adolescents and young adults aged 10–24 years from 1990 to 2019: A trend analysis based on the global burden of disease study 2019. Asian J Psychiatr. 2024;92:103905. 38262303 10.1016/j.ajp.2023.103905

[2] Lundberg J, Cars T, Lööv S, Söderling J, Tiihonen J, Leval A, et al. Clinical and societal burden of incident major depressive disorder: A population-wide cohort study in Stockholm. Acta Psychiatr Scand. 2022;146(1):51–63. 35165894 10.1111/acps.13414 PMC9310720

[3] Yan G, Zhang Y, Wang S, Yan Y, Liu M, Tian M, et al. Global, regional, and National Temporal trend in burden of major depressive disorder from 1990 to 2019: an analysis of the global burden of disease study. Psychiatry Res. 2024;337:115958. 38772160 10.1016/j.psychres.2024.115958

[4] Petta J, Falcao AL, Soares G, Lourenco A. Another tragic pandemic strikes: it is suicide. Eur Psychiatry. 2023;66:S1109–S.

[5] Ong MS, Lakoma M, Gees Bhosrekar S, Hickok J, McLean L, Murphy M, et al. Risk factors for suicide attempt in children, adolescents, and young adults hospitalized for mental health disorders. Child Adolesc Ment Health. 2021;26(2):134–42. 32569425 10.1111/camh.12400

[6] American Psychiatric A. Diagnostic and statistical manual of mental disorders. 5th ed. Arlington, VA: American Psychiatric Publishing; 2013.

[7] Suppes T, Ostacher M. Mixed features in major depressive disorder: diagnoses and treatments. CNS Spectr. 2017(1092–8529 (Print)). 10.1017/S1092852917000256 28462772

[8] Zimmerman M. How many criteria should be required to define the DSM-5 mixed features specifier in depressed patients? Eur Psychiatry. 2024;67(S1):S298–S. 10.4088/JCP.24m15406 41603782

[9] Na KS, Kang JM, Cho SE. Prevalence of DSM-5 mixed features: A meta-analysis and systematic review. J Affect Disord. 2021;282:203–10. 33418368 10.1016/j.jad.2020.12.149

[10] Hasin DS, Sarvet AL, Meyers JL, Saha TD, Ruan WJ, Stohl M, et al. Epidemiology of adult DSM-5 major depressive disorder and its specifiers in the united States. JAMA Psychiatry. 2018;75(4):336–46. 29450462 10.1001/jamapsychiatry.2017.4602 PMC5875313

[11] Shim IH, Woo YS, Bahk WM. Prevalence rates and clinical implications of bipolar disorder with mixed features as defined by DSM-5. J Affect Disord. 2015;173:120–5. 25462405 10.1016/j.jad.2014.10.061

[12] Sverdlichenko I, Jansen K, Souza LDM, da Silva RA, Kapczinski F, Cardoso TA. Mixed episodes and suicide risk: a community sample of young adults. J Affect Disord. 2020(1573–2517 (Electronic)). 10.1016/j.jad.2020.01.111 32056885

[13] Fiedorowicz JG, Persons JE, Assari S, Ostacher MJ, Goes FS, Nurnberger JI, et al. Moderators of the association between depressive, manic, and mixed mood symptoms and suicidal ideation and behavior: an analysis of the National network of depression centers mood outcomes program. J Affect Disord. 2021;281:623–30. 33234283 10.1016/j.jad.2020.11.101 PMC7855874

[14] Peyre H, Hoertel N, Pignon B, Amad A, Roelandt JL, Benradia I et al. Mixed features and nonfatal suicide attempt among individuals with major depressive episode: insights from the French MHGP survey. J Clin Psychiatry. 2024(1555–2101 (Electronic)). 10.4088/JCP.24m15445 39508717

[15] Zajac IT, Rice S, Proeve M, Kealy D, Oliffe JL, Ogrodniczuk JS. Suicide risk, psychological distress and treatment preferences in men presenting with prototypical, externalising and mixed depressive symptomology. J Ment Health. 2022;31(3):309–16. 32401094 10.1080/09638237.2020.1755026

[16] Sani G, Napoletano F, Vöhringer PA, Sullivan M, Simonetti A, Koukopoulos A, et al. Mixed depression: clinical features and predictors of its onset associated with antidepressant use. Psychother Psychosom. 2014;83(4):213–21. 24970376 10.1159/000358808

[17] Frazier EA, Swenson LP, Mullare T, Dickstein DP, Hunt JI. Depression with mixed features in adolescent psychiatric patients. Child Psychiatry Hum Dev. 2017;48(3):393–9. 27349656 10.1007/s10578-016-0666-z

[18] Zheng Y, Zhao J, Phillips M, Liu J, Cai M, Sun S, et al. Validity and reliability of the Chinese Hamilton depression rating scale. Br J Psychiatry. 1988;152(5):660–4. 3167442 10.1192/bjp.152.5.660

[19] Yin L, Song TH, Wei YY, Zhang LG, Zhou SJ, Yu JJ, et al. Relationship between affective temperaments and suicide risk in patients with First-Onset major depressive disorder. Front Psychiatry. 2022;13:893195. 35747102 10.3389/fpsyt.2022.893195 PMC9211372

[20] Ma X, Cao J, Zheng H, Mei X, Wang M, Wang H, et al. Peripheral body temperature rhythm is associated with suicide risk in major depressive disorder: a case-control study. Gen Psychiatr. 2021;34(1):e100219. 33644687 10.1136/gpsych-2020-100219 PMC7871238

[21] Buuren S, Groothuis-Oudshoorn K. MICE: multivariate imputation by chained equations in R. J Stat Softw. 2011;45:1–67.

[22] Austin PC, White IR, Lee DS, van Buuren S. Missing data in clinical research: A tutorial on multiple imputation. Can J Cardiol. 2021;37(9):1322–31. 33276049 10.1016/j.cjca.2020.11.010 PMC8499698

[23] Rubin DB. Multiple imputation for nonresponse in surveys. New York: Wiley; 1987.

[24] Collett D. Modelling survival data in medical research. Boca Raton: CRC; 2015.

[25] Bollen KA, Curran PJ. Latent curve models: A structural equation perspective. Hoboken, NJ: Wiley; 2006.

[26] Andersen PK, Borgan Ø, Gill RD, Keiding N. Statistical models based on counting processes. New York: Springer; 1993.

[27] McGinty J, Sayeed Haque M, Upthegrove R. Depression during first episode psychosis and subsequent suicide risk: A systematic review and meta-analysis of longitudinal studies. Schizophr Res. 2018;195:58–66. 28982553 10.1016/j.schres.2017.09.040

[28] Hawton K, Sutton L, Haw C, Sinclair J, Harriss L. Suicide and attempted suicide in bipolar disorder: a systematic review of risk factors. J Clin Psychiatry. 2005;66(6):693–704. 15960561 10.4088/jcp.v66n0604

[29] Eberhard J, Weiller E. Suicidality and symptoms of anxiety, irritability, and agitation in patients experiencing manic episodes with depressive symptoms: a naturalistic study. Neuropsychiatr Dis Treat. 2016;12:2265–71. 27621637 10.2147/NDT.S111094 PMC5012613

[30] Muneer A. Mixed States in bipolar disorder: Etiology, pathogenesis and treatment. Chonnam Med J. 2017;53(1):1–13. 28184334 10.4068/cmj.2017.53.1.1 PMC5299125

[31] Sheppler C, Edelmann A, Firemark A, Sugar C, Lynch F, Dickerson J et al. Stepped care for suicide prevention in teens and young adults: design and methods of a randomized controlled trial. Contemp Clin Trials. 2022:106959. 10.1016/j.cct.2022.106959 PMC10832890 36228984

[32] Richards JA, Cruz M, Stewart C, Lee A, Ryan T, Ahmedani B, et al. Effectiveness of integrating suicide care in primary care: secondary analysis of a Stepped-Wedge, cluster randomized implementation trial. Annals of internal medicine; 2024. 10.7326/M24-0024 PMC12005173 39348695

[33] First MB, Williams JBW, Karg RS, Spitzer RL. Structured Clinical Interview for DSM-5^®^ Disorders, Clinician Version (SCID-5-CV). Arlington, VA: American Psychiatric Association Publishing; 2016. p. 94.

[34] Kuo WH, Gallo JJ, Eaton WW. Hopelessness, depression, substance disorder, and suicidality–a 13-year community-based study. Soc Psychiatry Psychiatr Epidemiol. 2004;39(6):497–501. 15205735 10.1007/s00127-004-0775-z

[35] Abreu LN, Oquendo MA, Galfavy H, Burke A, Grunebaum MF, Sher L, et al. Are comorbid anxiety disorders a risk factor for suicide attempts in patients with mood disorders? A two-year prospective study. Eur Psychiatry. 2018;47:19–24. 29096128 10.1016/j.eurpsy.2017.09.005 PMC5766396

[36] Jones AA, Hard G, Gray J, Apsley HB, Santos-Lozada AR. The role of substance use disorders on suicidal Ideation, Planning, and attempts: A nationally representative study of adolescents and adults in the united States, 2020. Subst Abuse. 2023;17:11782218231216233. 38115827 10.1177/11782218231216233 PMC10729622

[37] Dube SR, Anda RF, Felitti VJ, Chapman DP, Williamson DF, Giles WH. Childhood abuse, household dysfunction, and the risk of attempted suicide throughout the life span: findings from the adverse childhood experiences study. JAMA. 2001;286(24):3089–96. 11754674 10.1001/jama.286.24.3089

[38] Vidal-Ribas P, Janiri D, Doucet GE, Pornpattananangkul N, Nielson DM, Frangou S, et al. Multimodal neuroimaging of suicidal thoughts and behaviors in a U.S. Population-Based sample of School-Age children. Am J Psychiatry. 2021;178(4):321–32. 33472387 10.1176/appi.ajp.2020.20020120 PMC8016742

[39] Klonsky ED, Saffer BY, Bryan CJ. Ideation-to-action theories of suicide: a conceptual and empirical update. Curr Opin Psychol. 2018;22:38–43. 30122276 10.1016/j.copsyc.2017.07.020

[40] Klonsky ED, May AM. The Three-Step Theory (3ST): a new theory of suicide rooted in the ideation-to-action framework. Int J Cogn Ther. 2015;8(2):114 – 29.

