# Supplementary material for: Mixed features and suicide attempts in youth depression: a six-month follow-up study
Source: BMC Psychiatry. 2026 Jan 14;26:134. doi: 10.1186/s12888-026-07783-x (PMC12888115; doi:10.1186/s12888-026-07783-x)
Supplement: Supplementary file 1 — Supplementary Material 1 [file 12888_2026_7783_MOESM1_ESM.docx]

| **Supplementary Table. Logistic Regression Analyses of Baseline Predictors of Missingness at 1-, 3-, and 6-Month Follow-ups.** | | | | | | |
| --- | --- | --- | --- | --- | --- | --- |
|  | 1-month | | 3-month | | 6-month | |
| Variables | OR (95% CI) | P value | OR (95% CI) | P value | OR (95% CI) | P value |
| Gender (Ref = Male) |  |  |  |  |  |  |
| Female | 0.69 (0.48-0.97) | 0.034 | 1.05 (0.74-1.47) | 0.798 | 0.80 (0.58-1.12) | 0.191 |
| Episode Status (Ref = First Episode) |  |  |  |  |  |  |
| Recurrent episode | 1.01 (0.71-1.44) | 0.953 | 1.25 (0.89-1.76) | 0.195 | 1.14 (0.81-1.59) | 0.450 |
| Smoke history(Ref = No) |  |  |  |  |  |  |
| Yes | 0.76 (0.47-1.24) | 0.270 | 1.01 (0.65-1.57) | 0.970 | 0.99 (0.64-1.52) | 0.951 |
| Alcohol history(Ref = Yes) |  |  |  |  |  |  |
| No | 1.27 (0.69-2.35) | 0.448 | 0.72 (0.42-1.23) | 0.234 | 0.82 (0.48-1.41) | 0.470 |
| Family history(Ref = No) |  |  |  |  |  |  |
| Yes | 0.84 (0.58-1.22) | 0.352 | 1.03 (0.73-1.45) | 0.866 | 0.95 (0.68-1.33) | 0.751 |
| HAMD17 no suicide | 1.00 (0.97-1.02) | 0.939 | 1.02 (1.00-1.05) | 0.068 | 1.04 (1.02-1.07) | <0.001 |
| HAMD17 suicide | 1.02 (0.90-1.16) | 0.749 | 0.98 (0.86-1.10) | 0.697 | 0.93 (0.83-1.05) | 0.252 |
| Sucide History (Ref = No) |  |  |  |  |  |  |
| Yes | 1.16 (0.84-1.60) | 0.358 | 0.97 (0.71-1.32) | 0.846 | 0.91 (0.67-1.23) | 0.545 |
| Age | 0.89 (0.85-0.94) | <0.001 | 0.92 (0.88-0.97) | <0.001 | 0.84 (0.81-0.88) | <0.001 |
| Illness duration(Ref = < 6 months) |  |  |  |  |  |  |
| 6 – 24 months | 1.18 (0.78-1.79) | 0.432 | 0.88 (0.60-1.30) | 0.526 | 0.56 (0.38-0.83) | 0.004 |
| > 24 months | 1.08 (0.67-1.74) | 0.746 | 0.77 (0.49-1.21) | 0.255 | 0.62 (0.40-0.96) | 0.034 |
| Note. Odds ratios (OR) and 95% confidence intervals (CI) were derived from logistic regression models examining whether baseline demographic and clinical characteristics predicted missingness at each follow-up time point (1-, 3-, and 6-month). An OR greater than 1 indicates a higher likelihood of missing data for that variable category. | | | | | | |

**Supplementary Figure. Missing Data Patterns for Suicide Attempt Variables Across Follow-Up Time Points.**


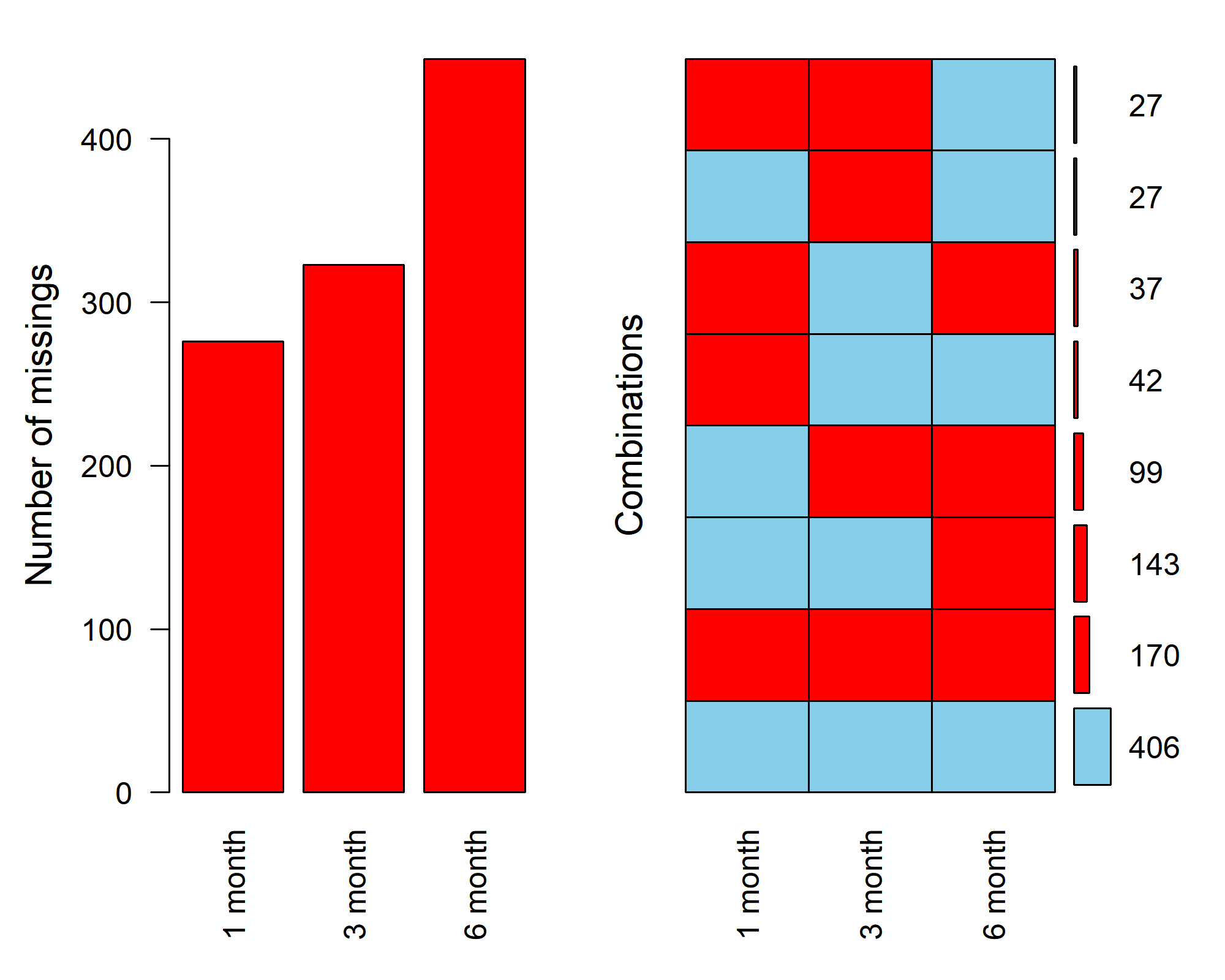


Note. Visualization of missing data patterns for suicide attempt variables at 1-, 3-, and 6-month follow-up. Bar heights indicate the number of missing (red) and observed (blue) values at each time point. The left panel summarizes the number of missing observations for each follow-up assessment, while the right panel shows combinations of missingness across follow-up time points.
